# Comparative Evaluation of Tetracycline Hydrochloride Fiber and Simvastatin Gel as an Adjunct to Scaling and Root Planing in Periodontitis Patients

**DOI:** 10.7759/cureus.42314

**Published:** 2023-07-22

**Authors:** Samidha Jambhekar, Mrunmayee Soman, Ratika Shrivastava, Roja Ventrapragada, Shweta Sarate, Tejaswi Kodem

**Affiliations:** 1 Department of Periodontics, Dr. DY Patil Dental College and Hospital, Mumbai, IND; 2 Department of Dentistry, Dr. DY Patil Dental College and Hospital, Pimpri, IND; 3 Department of Periodontology, Rishiraj College of Dental Science & Research Center, Bhopal, IND; 4 Department of Periodontology, Partha Dental Skin Hair Clinic, Mangalagiri, IND; 5 Department of Periodontology, People's College of Dental Sciences, Bhopal, IND; 6 Department of Periodontology, Gitam Dental College and Hospital, Visakhapatnam, IND

**Keywords:** srp, local drug delivery agents, periodontitis, simvastatin gel, tetracycline fibers

## Abstract

Inflammation of oral soft tissues, caused by periodontal disease, results in the loss of attachment to supporting therapy and is a severe threat to dental health. Although there are a number of therapeutic options available, mechanical debridement continues to be the gold standard. Scaling and root planing is the gold standard therapy for periodontitis, but this research aims to examine the efficacy of tetracycline fibers and simvastatin gel as local drug delivery methods.

We evaluated 60 sites, splitting them into three groups: 20 sites received just scaling and root planing; 20 sites received scaling and root planing plus simvastatin gel; and 20 sites received scaling and root planing plus tetracycline fibers. Clinical indicators such as the gingival index, the modified sulcular bleeding index, and the probing depth were measured at the start of the study, after one week, after one month, after three months, and after six months.

After six months, the simvastatin group reduced the gingival index and modified sulcular bleeding index more than the tetracycline group, whereas the tetracycline group reduced probing depth more than the simvastatin group.

## Introduction

Antibacterial agents have occasionally been used, with varying degrees of success [1]. Poor response to antibacterial therapy has been linked to insufficient drug concentrations reaching the site of action or insufficient drug levels being sustained for long enough [1]. Anti-infective measures that target the pathogenic organisms present in dental plaque on the tooth surface are crucial to the treatment's success.

Traditionally, the primary means of therapy for periodontal disease includes mechanical debridement [1]. The complex anatomy of the root and the contours of the lesion may hamper the treatment and prevent sufficient reduction of the bacterial load. Systemically administered antibacterial agents are able to achieve relatively low concentrations in pockets even at high dosages [2]. Moreover, unwanted effects, such as gastrointestinal disturbances and the development of antibiotic resistance, cannot be totally ruled out. To overcome these limitations, local drug delivery was introduced [1].

Some pathognomic organisms are resistant to mechanical or electrically driven tools but may be reduced or eradicated when placed in direct touch with an antibiotic or antiseptic applied to the root surface[1]. Treatment for periodontal disease has included the use and study of a wide range of antimicrobials, including tetracycline hydrochloride, doxycycline, minocycline, metronidazole, statins, chlorhexidine, etc., for local drug delivery [3].

In addition to traditional periodontal therapy (scaling and root planing), this research will evaluate the efficacy of tetracycline fibers and simvastatin gel as local medication delivery methods[2].

## Materials and methods

Patients were chosen from a periodontics clinic's outpatient population. The biostatistician's support in determining the sample size and the study's approval by the Institutional Review Board and Ethical Committee (reference number: 049, dated 07/12/2015) are both cited as sources of credibility. Patients were given information about the trial before giving their agreement to participate.

A total of 20 patients with periodontitis and probing depths of less than 5 mm in at least three molars from various quadrants were chosen. Three sites in each patient with periodontal pockets, less than 5 mm wide, were selected, where one site represented the tooth's mesial and distal aspects. These sites were randomly distributed by the use of convenient sampling by a subject expert by the use of a computer-generated list into the following three groups: Group I - periodontal pockets were treated with scaling and root planing alone (control group: 20 sites); Group II - periodontal pockets were treated with simvastatin gel as an adjunct to scaling and root planing (simvastatin group: 20 sites); and Group III - periodontal pockets were treated with tetracycline fibers (Periodontal AB Plus, Advanced Biotech, India) as an adjunct to scaling and root planing (tetracycline group: 20 sites).

Inclusion criteria for patient selection: Systemically healthy patients and patients with periodontitis and having probing depth ≥ 5 mm in molar teeth were included in the study.

Exclusion criteria for patient selection: Excluded from the trial were individuals with known allergies to tetracycline and simvastatin, those who had taken antibiotics within the two weeks before enrollment, those who were smokers or alcoholics, those who had impaired immune systems, and pregnant or nursing women. The materials used were tetracycline fibers and simvastatin gel; tetracycline fibers, which are commercially available, were made up of 2 mg of tetracycline hydrochloride in 25 mg of collagen fibrils, and 100 g of simvastatin gel was prepared under supervision at the Department of Pharmaceutics, College of Pharmacy.

Gel preparation: 1 gram of Carbopol was soaked in 75 ml of distilled water for two hours and continuously stirred by using a magnetic stirrer to form a polymer solution, and 1 ml of triethanolamine was added to this polymer solution to get a gel consistency. The gel formed was kept aside, and another solution was prepared by dissolving 1.2 grams of simvastatin powder in 5 grams of propylene glycol. The solution formed by propylene glycol and simvastatin was added to the polymer solution in small proportion with continuous stirring until a homogenous preparation was achieved. The final weight of the preparation was adjusted to 100 grams by adding a sufficient amount of distilled water. Prepared simvastatin gel was stored in an airtight container made of borosilicate glass in a dry and cool place.

**Figure 1:**
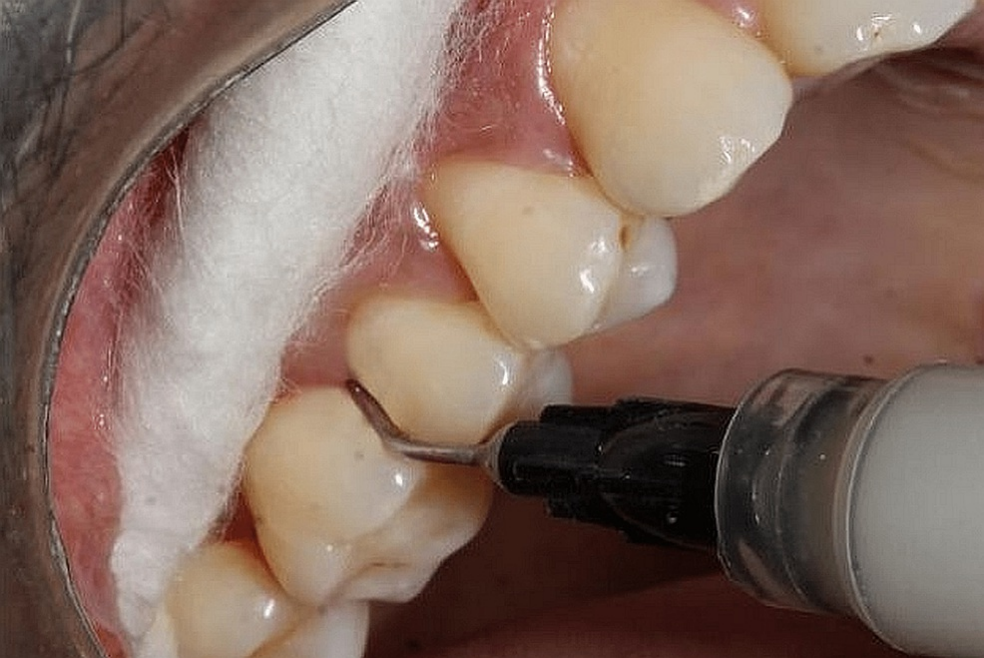
Simvastatin gel insertion in the pocket

In all, 60 locations were examined. Full-mouth scaling and root planing was performed on the chosen patients. The first location (Group I) just underwent scaling and root planing. Group II received an additional simvastatin gel treatment at the second location, delivered by a syringe with a blunt cannula. Due to the produced formulation of simvastatin gel increasing in viscosity upon insertion in the pocket, leading to swelling and occlusion of the periodontal pockets, no periodontal dressing was used upon delivery [3] (Figure 1).

**Figure 2:**
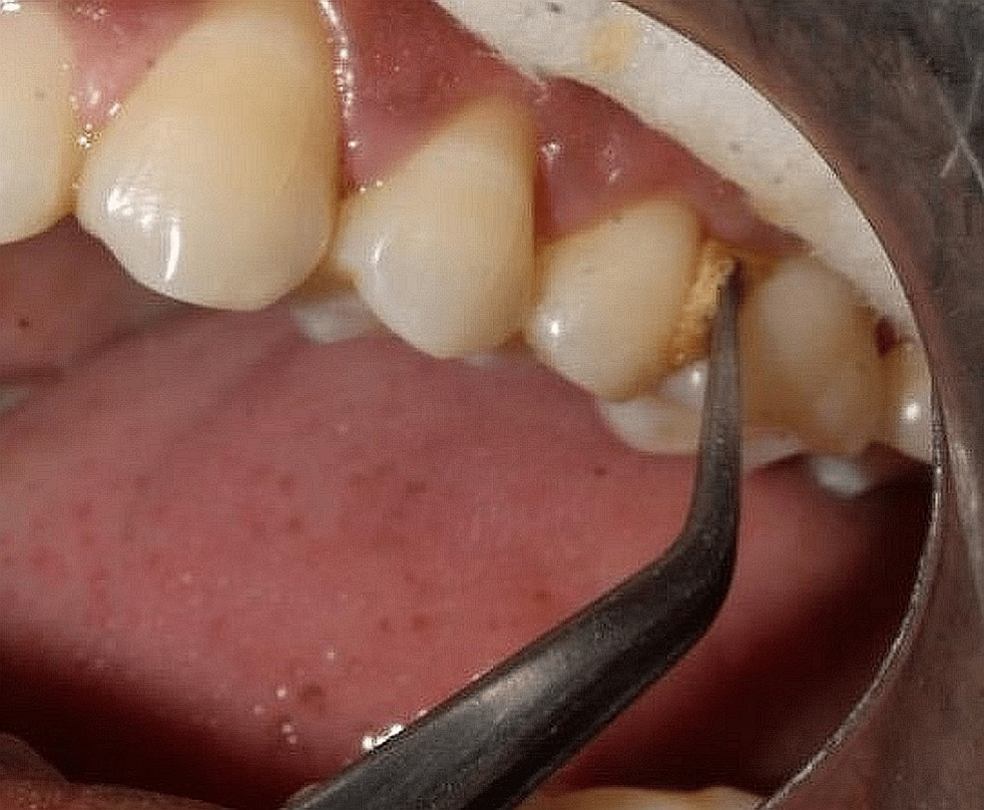
Tetracycline fibers placement in the pocket

The third site was treated with tetracycline fibers inserted within the periodontal pockets until it was up to approximately 1 mm apical to the gingival margin (Group III). The periodontal dressing was placed [4] (Figure 2).

**Figure 3:**
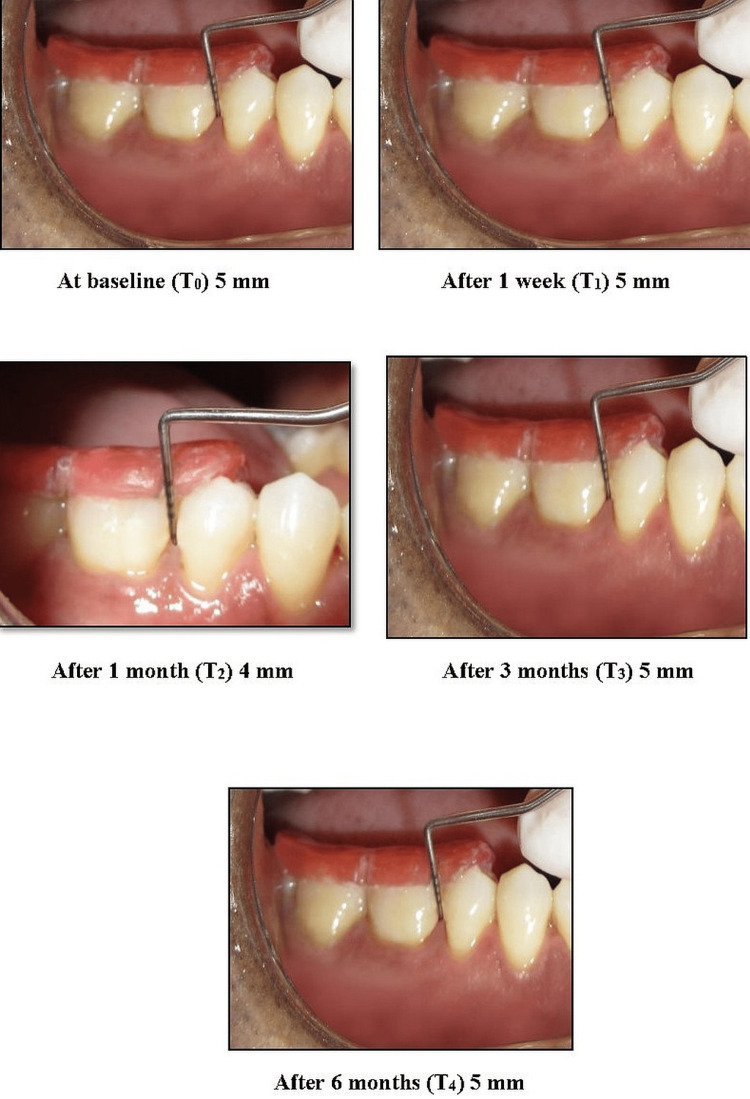
Pocket depth measurements at different intervals of time

Clinical parameters, such as gingival index, modified sulcular bleeding index, and probing depth of the selected sites, were assessed at T0 (baseline), T1 (on the seventh day), T2 (after one month), T3 (after three months), and T4 (after six months) (Figure 3).

Patients were also told to avoid brushing the region surrounding the treatment for seven days and to avoid biting on hard or sticky foods. On the seventh day, the periodontal dressing was removed.

## Results

Gingival index

Differences in the gingival index between the control group, simvastatin group, and tetracycline group from baseline to six months were determined using the ANOVA test. At baseline, the mean gingival index of the control group (2.2000), simvastatin group (2.4500), and tetracycline group (2.3500) was compared and exhibited readings that were not statistically significant. Gingival index values decreased after one week in all three groups, although there was no statistically significant difference between them (control group: 1.4000; simvastatin group: 1.3000; tetracycline group: 1.2500). Statistically significant differences between the control group (1.250), the simvastatin group (900), and the tetracycline group (900) were seen after one month of treatment. The mean gingival index at three months was 0.8000 in the tetracycline group, 0.5500 in the simvastatin group, and 1.2500 in the control group. A statistically significant result was revealed by comparing the control group (1.2000), simvastatin group (0.5000), and tetracycline group (0.6500) after six months.

In the control group, a highly statistically significant reduction was seen in the mean gingival index from baseline to after six months. A similar pattern of reduction was seen in the gingival index within the simvastatin group and within the tetracycline group when assessed at different time intervals (Table 1).

**Table 1:** Difference in the gingival index between the control, simvastatin, and tetracycline groups at different time intervals using the ANOVA test

Time interval	Groups	N	Mean	Standard Deviation	95% Confidence Interval		F	p
					Lower	Upper		
T_0_	Control	20	2.2000	0.76777	1.8407	2.5593	0.588	0.559
	Simvastatin	20	2.4500	0.68633	2.1288	2.7712		
	Tetracycline	20	2.3500	0.74516	2.0013	2.6987		
T_1_	Control	20	1.4000	0.50262	1.1648	1.6352	0.292	0.748
	Simvastatin	20	1.3000	0.65695	0.9925	1.6075		
	Tetracycline	20	1.2500	0.71635	0.9147	1.5853		
T_2_	Control	20	1.2500	0.44426	1.0421	1.4579	4.979	0.010
	Simvastatin	20	0.9000	0.30779	0.7559	1.0441		
	Tetracycline	20	0.9000	0.44721	0.6907	1.1093		
T_3_	Control	20	1.2500	0.63867	0.9511	1.5489	8.014	0.001
	Simvastatin	20	0.5500	0.51042	0.3111	0.7889		
	Tetracycline	20	0.8000	0.52315	0.5552	1.0448		
T_4_	Control	20	1.2000	0.61559	0.9119	1.4881	8.259	0.001
	Simvastatin	20	0.5000	0.60698	0.2159	0.7841		
	Tetracycline	20	0.6500	0.48936	0.4210	0.8790		

Modified sulcular bleeding index

The modified sulcular bleeding index of the control group (2.6000), simvastatin group (2.6500), and tetracycline group (2.6000) when compared at baseline was not statistically significant. After one week, a statistically significant difference was seen among the control group (2.0500), simvastatin group (1.5000), and tetracycline group (1.6000). On comparison between the control group, simvastatin group, and tetracycline group, the modified sulcular bleeding index was further changed to 2.0500, 1.0000, and 1.3500, respectively, after one month, which was highly statistically significant. When all three groups were analyzed and compared, after three and six months showed a highly statistically significant outcome in the mean modified sulcular bleeding index: 1.8000 and 2.0000 in the control group, respectively; 0.5000 and 0.2500 in the simvastatin group, respectively; and 0.7000 and 0.4000 in the tetracycline group, respectively.

The difference in the modified sulcular bleeding index at different time intervals within the control group was determined using repeated measures of the ANOVA test. In the control group, the mean modified sulcular bleeding index was significantly reduced from baseline to after three months and slightly increased after six months. In the simvastatin group from baseline to after six months, a highly significant reduction was seen in the modified sulcular bleeding index. A similar framework was observed in the tetracycline group when assessed from baseline to after six months (Table 2).

**Table 2:** Difference in the modified sulcular bleeding index between the control, simvastatin, and tetracycline groups at different time intervals using the ANOVA test

Time interval	Groups	N	Mean	Standard Deviation	95% Confidence Interval		F	p
					Lower	Upper		
T_0_	Control	20	2.6000	0.50262	2.3648	2.8352	0.067	0.935
	Simvastatin	20	2.6500	0.48936	2.4210	2.8790		
	Tetracycline	20	2.6000	0.50262	2.3648	2.8352		
T_1_	Control	20	2.0500	0.51042	1.8111	2.2889	6.634	0.003
	Simvastatin	20	1.5000	0.51299	1.2599	1.7401		
	Tetracycline	20	1.6000	0.50262	1.3648	1.8352		
T_2_	Control	20	2.0500	0.68633	1.7288	2.3712	13.866	0.001
	Simvastatin	20	1.0000	0.56195	0.7370	1.2630		
	Tetracycline	20	1.3500	0.67082	1.0360	1.6640		
T_3_	Control	20	1.8000	0.52315	1.5552	2.0448	30.359	0.001
	Simvastatin	20	0.5000	0.51299	0.2599	0.7401		
	Tetracycline	20	0.7000	0.65695	0.3925	1.0075		
T_4_	Control	20	2.0000	0.85840	1.5983	2.4017	47.563	0.001
	Simvastatin	20	0.2500	0.44426	0.0421	0.4579		
	Tetracycline	20	0.4000	0.50262	0.1648	0.6352		

Pocket depth

At baseline, the mean pocket depth of control group was 5.1250, the simvastatin group was 5.0750, and the tetracycline group was 5.1750; when compared, all these three groups were not statistically significant. There was a slight difference in the mean pocket depth after one week in the control group (4.1000), simvastatin group (3.6000), and tetracycline group (3.7250); however, it was not statistically significant. All three groups were evaluated and compared after one month, which showed a highly statistically significant change among the control group (3.6250), simvastatin group (3.5250), and tetracycline group (2.8000). A statistically significant outcome was observed after three months, when comparing the control group (3.7750), simvastatin group (3.4500), and tetracycline group (3.1750). The P value (0.037) was statistically significant after three months. On comparison, all three groups showed (control group: 3.8000, simvastatin group: 3.5500, and tetracycline group: 2.6000) a highly statistically significant difference after six months.

In the controlgroup, a highly statistically significant reduction was seen in pocket depth from baseline to one week and further reduced at one month. There was a little improvement at three and six months, but the p-value was very significant. A large decrease was seen in the simvastatin group from baseline to six months, followed by a little rise at six months. From baseline to one month, the tetracycline group demonstrated a very significant decrease in pocket depth, which was followed by a modest rise at three months and a further decrease at six months (Table 3).

**Table 3:** Difference in pocket depth between the control, simvastatin, and tetracycline groups at different time intervals using the ANOVA test

Time interval	Groups	N	Mean	Standard Deviation	95% Confidence Interval		F	p
					Lower	Upper		
T_0_	Control	20	5.1250	0.45523	4.9119	5.3381	0.279	0.758
	Simvastatin	20	5.0750	0.18317	4.9893	5.1607		
	Tetracycline	20	5.1750	0.54471	4.9201	5.4299		
T_1_	Control	20	4.1000	0.55251	3.8414	4.3586	2.424	0.098
	Simvastatin	20	3.6000	0.80459	3.2234	3.9766		
	Tetracycline	20	3.7250	0.85031	3.3270	4.1230		
T_2_	Control	20	3.6250	0.58208	3.3526	3.8974	10.564	0.001
	Simvastatin	20	3.5250	0.73404	3.1815	3.8685		
	Tetracycline	20	2.8000	0.52315	2.5552	3.0448		
T_3_	Control	20	3.7750	0.63815	3.4763	4.0737	3.507	0.037
	Simvastatin	20	3.4500	0.87208	3.0419	3.8581		
	Tetracycline	20	3.1750	0.61291	2.8881	3.4619		
T_4_	Control	20	3.8000	0.54772	3.5437	4.0563	12.890	0.001
	Simvastatin	20	3.5500	1.16867	3.0030	4.0970		
	Tetracycline	20	2.6000	0.44721	2.3907	2.8093		

## Discussion

The bacterial biofilm and the immune-inflammatory response of the host interact in intricate ways to cause periodontal diseases. Despite the fact that mechanical debridement is still the backbone of the treatment and the patient's strict maintenance of dental hygiene is essential to its effectiveness, regions inaccessible to periodontal instrumentation remain a worry. Due to their hiding places inside the gingival and dental tissues or elsewhere inaccessible to periodontal devices, pathogenic microorganisms may be resistant to mechanical debridement alone. A microbial shift occurs after subgingival debridement without supplementary antibacterial treatments. Three months after the mechanical debridement, the beneficial effect of instrumentation completely disappears [5]. Further, repeated scaling and root planing can introduce a Schwartzman-like reaction [6]. It has been shown via the use of checkerboard DNA-based detection that scaling and root planing alone often does not result in the elimination of important periodontal infections.

Due to its microbial origin, periodontitis often requires the supplementary use of antibiotics, either systemically or topically. The use of a local drug-delivery system was motivated by the many drawbacks of systemic antibiotic therapy, such as hypersensitivity reaction, organ toxicity, and the development of resistant bacteria, as well as the need for higher dosages to attain the necessary concentration of antibiotics in the gingival crevicular fluid at the target site [7].

Therapeutic agents may be delivered directly to the locations where they are needed to treat illness, minimizing systemic absorption and the likelihood of unwanted side effects, owing to local drug delivery systems. When compared to systemic administration, local medication delivery may result in a target site concentration that is up to 10 times greater. Many different agents have been tried for medication delivery at a specific site.

Hypercholesterolemia, hyperlipidemia, and atherosclerosis are all treatable conditions that benefit from simvastatin's ability to block the enzyme 3-hydroxy-3-methyl-glutaryl coenzyme A reductase by competitive inhibition [8]. Carbopol was used to create a simvastatin gel for this investigation. Carbopol polymers are characterized by their high viscosity at low concentrations, distinctive flow behavior, compatibility with numerous active substances, bio-adhesive qualities, strong thermal stability, outstanding organoleptic features, and good patient acceptability [9].

Bone resorption may be stopped by preventing the enzyme HMG-CoA reductase from doing its job and thus blocking the mevalonate pathway. This prevents osteoclasts from forming their characteristic ruffled borders and vesicles, two processes crucial to their bone-resorbing function. This leads to a decrease in bone resorption and inactivation of osteoclasts. Statins also increase the expression of bone morphogenetic protein-2 in osteoblasts [10]. In preosteoblastic murine cells, simvastatin upregulates the production of vascular endothelial growth factor (VEGF), which aids in osteoblast differentiation and bone nodule development [11]. In the periodontal tissues, simvastatin decreased the expression of iNOS, MMP1, MMP-8, RANK, and RANKL [12]. Through the activation of the ERK1/2 pathway, it promotes periodontal ligament cell development toward an osteoblastic phenotype [13]. Because of its hydrophobic nature, simvastatin is an effective antibiotic against both *Actinobacillus actinomycetemcomitans* and *Porphyromonas gingivalis*, hastening the death of microbial cells via apoptosis, and distressing the bacterial membrane in a "soap-like" way [14].

Tetracycline fibers, first found in 1948 as the fermentation products of a soil bacterium called *Streptomyces aureofaciens*, were also utilized as an experimental agent in the current investigation [15]. Microorganisms such as *Bacteroides gingivalis* and *Actinobacillus actinomycetemcomitans*, which are thought to be periodontopathogens, are present at a concentration much higher in the gingival crevicular fluid at the site of the lesion than in serum. Tetracycline has the ability to suppress these microorganisms [16]. Tetracyclines' ability to lower oxidative stress markers also lessens inflammation when used adjunctively [17].

Tetracyclines prevent the degradation of connective tissue via many methods. Collagenases and gelatinases are both blocked by tetracycline and its derivatives [18]. Tetracyclines circulate largely as Ca++ and Mg++ chelates in the blood plasma because they bind divalent metal cations. The biological significance of their function as calcium ionophores cannot be overstated. The therapeutic potential exists for the use of tetracyclines due to their antiangiogenic activity, which inhibits the growth of new blood vessels during inflammatory processes [19]. 

Tetracycline has multiple abilities, such as chelate cations; it not only suppresses prostaglandin E2 (PGE2) and endotoxin-stimulated bone resorption but also inhibits resorption stimulated by PTH. Along with their anti-inflammatory, antimicrobial, non-antimicrobial properties, tetracyclines have the ability to promote fibroblast and connective tissue to tooth surfaces, which is relevant to the regeneration of periodontal tissues lost during diseases [20]. Within the good aspects of this research study, there are a few shortcomings, such as the small sample size. Thus, the evaluation of simvastatin's effect on bone regeneration by radiographs and biomarkers needs to be assessed, and other locally delivered agents have to be evaluated.

## Conclusions

When used in conjunction with scale and root planing, simvastatin gel or tetracycline fibers significantly improved all clinical measures. When comparing the decrease in the gingival index, the modified sulcular bleeding index, and pocket depth between the experimental group and the control group (scaling and root planing alone), the experimental group, simvastatin gel plus tetracycline fibers, performs very well. Reductions in the gingival index and modified sulcular bleeding index were more pronounced in the simvastatin group than in the tetracycline group, whereas reductions in probing depth were more pronounced in the tetracycline fiber group than in the simvastatin group. When all of these factors are considered, non-surgical periodontal therapy with tetracycline fibers or simvastatin gel may be a viable alternative to surgical treatment for mild to moderate periodontitis.

## References

[1] Goodson JM, Cugini MA, Kent RL (1991). Multicenter evaluation of tetracycline fiber therapy: II. Clinical response. J Periodontal Res.

[2] Drisko CL, Cobb CM, Killoy WJ (1995). Evaluation of periodontal treatments using controlled-release tetracycline fibers: clinical response. J Periodontol.

[3] Pradeep AR, Thorat MS (2010). Clinical effect of subgingivally delivered simvastatin in the treatment of patients with chronic periodontitis: a randomized clinical trial. J Periodontol.

[4] Sinha S, Kumar S, Dagli N, Dagli RJ (2014). Effect of tetracycline HCl in the treatment of chronic periodontitis - a clinical study. J Int Soc Prev Community Dent.

[5] Quirynen M, Teughels W, De Soete M, van Steenberghe D (2002). Topical antiseptics and antibiotics in the initial therapy of chronic adult periodontitis: microbiological aspects. Periodontol 2000.

[6] Farman M, Joshi RI (2008). Full-mouth treatment versus quadrant root surface debridement in the treatment of chronic periodontitis: a systematic review. Br Dent J.

[7] Gunjiganur Vemanaradhya G, Emani S, Mehta DS, Bhandari S (2017). Effect of 1.2% of simvastatin gel as a local drug delivery system on gingival crevicular fluid interleukin-6 & interleukin-8 levels in non surgical treatment of chronic periodontitis patients. Arch Oral Biol.

[8] Hunninghake DB (1998). Therapeutic efficacy of the lipid-lowering armamentarium: the clinical benefits of aggressive lipid-lowering therapy. Am J Med.

[9] Islam MT, Rodríguez-Hornedo N, Ciotti S, Ackermann C (2004). Rheological characterization of topical carbomer gels neutralized to different pH. Pharm Res.

[10] Kinra P, Khan S (2011). Simvastatin: its potential new role in periodontal regeneration. Biol Med.

[11] Dalcico R, de Menezes AM, Deocleciano OB, Oriá RB, Vale ML, Ribeiro RA, Brito GA (2013). Protective mechanisms of simvastatin in experimental periodontal disease. J Periodontol.

[12] Yazawa H, Zimmermann B, Asami Y, Bernimoulin JP (2005). Simvastatin promotes cell metabolism, proliferation, and osteoblastic differentiation in human periodontal ligament cells. J Periodontol.

[13] Ruan F, Zheng Q, Wang J (2012). Mechanisms of bone anabolism regulated by statins. Biosci Rep.

[14] Moon HJ, Kim SE, Yun YP, Hwang YS, Bang JB, Park JH, Kwon IK (2011). Simvastatin inhibits osteoclast differentiation by scavenging reactive oxygen species. Exp Mol Med.

[15] Emani S, Gunjiganur GV, Mehta DS (2014). Determination of the antibacterial activity of simvastatin against periodontal pathogens, Porphyromonas gingivalis and Aggregatibacter actinomycetemcomitans: an in vitro study. Contemp Clin Dent.

[16] Sapadin AN, Fleischmajer R (2006). Tetracyclines: nonantibiotic properties and their clinical implications. J Am Acad Dermatol.

[17] Golub LM, McNamara TF, D'Angelo G, Greenwald RA, Ramamurthy NS (1987). A non-antibacterial chemically-modified tetracycline inhibits mammalian collagenase activity. J Dent Res.

[18] Soory M (2008). A role for non-antimicrobial actions of tetracyclines in combating oxidative stress in periodontal and metabolic diseases: a literature review. Open Dent J.

[19] Golub LM, Lee HM, Ryan ME, Giannobile WV, Payne J, Sorsa T (1998). Tetracyclines inhibit connective tissue breakdown by multiple non-antimicrobial mechanisms. Adv Dent Res.

[20] Heyland DK, Johnson AP, Reynolds SC, Muscedere J (2011). Procalcitonin for reduced antibiotic exposure in the critical care setting: a systematic review and an economic evaluation. Crit Care Med.

